# Overview of the Development and Use of Akt Inhibitors in Prostate Cancer

**DOI:** 10.3390/jcm11010160

**Published:** 2021-12-29

**Authors:** Anis Gasmi, Guilhem Roubaud, Charles Dariane, Eric Barret, Jean-Baptiste Beauval, Laurent Brureau, Gilles Créhange, Gaëlle Fiard, Gaëlle Fromont, Mathieu Gauthé, Alain Ruffion, Raphaële Renard-Penna, Paul Sargos, Morgan Rouprêt, Guillaume Ploussard, Romain Mathieu

**Affiliations:** 1 Department of Urology, University of Rennes, 35000 Rennes, France; Romain.MATHIEU@chu-rennes.fr; 2 Department of Medical Oncology, Institut Bergonié, 33000 Bordeaux, France; G.Roubaud@bordeaux.unicancer.fr; 3 Department of Urology, Hôpital Européen Georges-Pompidou, AP-HP, Paris University, 75005 Paris, France; dcharlie8@hotmail.com; 4 Department of Urology, Institut Mutualiste Montsouris, 75005 Paris, France; Eric.Barret@imm.fr; 5 Department of Urology, La Croix du Sud Hôpital, Quint Fonsegrives, 31000 Toulouse, France; jbbeauval@gmail.com (J.-B.B.); g.ploussard@gmail.com (G.P.); 6 Department of Urology, CHU de Pointe-à-Pitre, University of Antilles, 97110 Pointe-à-Pitre, France; laurent.brureau@chu-guadeloupe.fr; 7 Department of Urology, University Hospital, Université Grenoble Alpes, 38000 Grenoble, France; gilles.crehange@curie.fr; 8 Department of Radiation Oncology, Curie Institute, 75005 Paris, France; gaellef@gmail.com; 9 Department of Pathology, CHRU Tours, 37000 Tours, France; gaelle.fromont-hankard@univ-tours.fr; 10 Department of Nuclear Medicine, Scintep, 38000 Grenoble, France; mathieugauthe@yahoo.fr; 11 Service d’Urologie Centre Hospitalier Lyon Sud, Hospices Civils de Lyon, 69000 Lyon, France; ruffion.alain@orange.fr; 12 Equipe 2, Centre d’Innovation en Cancérologie de Lyon (EA 3738 CICLY), Faculté de Médecine Lyon Sud, Université Lyon 1, 69000 Lyon, France; 13 Department of Radiology, Sorbonne University, AP-HP, Pitie-Salpetriere Hospital, 75013 Paris, France; raphaele.renardpenna@gmail.com; 14 Department of Radiotherapy, Institut Bergonié, 33000 Bordeaux, France; P.Sargos@bordeaux.unicancer.fr; 15 Department of Urology, Sorbonne University, GRC 5 Predictive Onco-Uro, AP-HP, Urology, Pitie-Salpetriere Hospital, 75013 Paris, France; mroupret@gmail.com; 16 IRSET (Institut de Recherche en Santé, Environnement et Travail), University of Rennes, Inserm, EHESP, 35000 Rennes, France

**Keywords:** prostate cancer, Akt, PTEN, castration resistance, Ipatasertib, Capivasertib

## Abstract

Deregulation of the PI3K-Akt-mTOR pathway plays a critical role in the development and progression of many cancers. In prostate cancer, evidence suggests that it is mainly driven by PTEN loss of function. For many years, the development of selective Akt inhibitors has been challenging. In recent phase II and III clinical trials, Ipatasertib and Capivasertib associated with androgen deprivation therapies showed promising outcomes in patients with metastatic castration-resistant prostate cancer and PTEN-loss. Ongoing trials are currently assessing several Akt inhibitors in prostate cancer with different combinations, at different stages of the disease.

## 1. Introduction

Akt protein, also known as protein kinase B (PKB), is at the crossroads of several signalling pathways. This protein plays a critical role in regulating diverse cellular functions including cell metabolism, proliferation, apoptosis suppression and angiogenesis. Alterations in the Akt-dependent pathways are associated with cancer, diabetes, cardiovascular and neurological diseases [1]. The upregulation of Akt has been reported in a variety of human malignancies, including digestive, neurological, gynaecological, and urological cancers [2]. Akt promotes cell survival and proliferation through its effects on the cellular growth factors and inhibits apoptosis through the inactivation of pro-apoptotic proteins [3]. Given these properties, there is a growing interest in developing anti-cancer drugs targeting this pathway. We aimed to describe, in this review, the PI3K-Akt pathway and the use of Akt inhibitors in prostate cancer.

## 2. PI3K-Akt-mTor Pathway Physiology and Drug Development

Akt is a cytosolic serine/threonine kinase that has three isoforms (Akt 1, 2 and 3). Structurally, it comprises three domains: a carboxyl-terminal, a central and an amino-terminal fragment. Akt isoforms are similar in their catalytic domains but diverge in the regulatory domain. Akt1 and Akt2 are ubiquitous, whereas Akt3 is predominantly found in the kidney, brain and heart. Their functions are partially overlapping but are distinct in cancer cells [4].

Akt, together with phosphoinositide 3-kinase (PI3K), are key elements of the AKT signalling cascade, which is also known as the PI3K/Akt pathway. It promotes growth and survival in response to extracellular stimuli. This signalling cascade can be activated by cytokine receptors, integrins, receptor tyrosine kinases, B and T cell receptors, G-protein-coupled receptors, and other signals [5].

**Figure 1:**
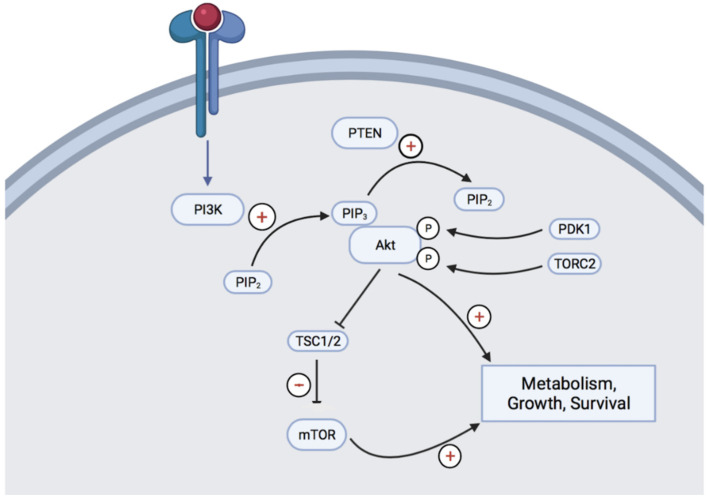
Overview of the PI3K-Akt-mTOR pathway.

Once activated, PI3K phosphorylates PIP2 (phosphatidylinositol 4,5-bisphosphates) to PIP3 (phosphatidylinositol (3,4,5)-trisphosphates), a reaction that is negatively regulated by PTEN (phosphatase and tensin homolog chromosome 10). PIP3 then recruits Akt and allows its phosphorylation through pyruvate dehydrogenase kinase 1 (PDK1) and mammalian target of rapamycin complex 2 (mTORC2). Then, Akt phosphorylates different membrane, cytosolic and nucleic proteins involved in cell growth and survival, among other cellular effects (Figure 1). Mammalian target of rapamycin (mTOR) is a downstream member of the Akt pathway and a key regulator of cell growth and metabolism [6].

PI3K: phosphoinositide 3-kinase, Akt: protein kinase B, PIP2: phosphatidylinositol 4,5-bisphosphates, PIP3: phosphatidylinositol (3,4,5)-trisphosphates, PTEN: phosphatase and tensin homolog chromosome 10, PDK1: pyruvate dehydrogenase kinase 1, mTORC2: mammalian target of rapamycin complex 2, TSC1/2: tuberous sclerosis proteins 1 and 2, mTOR: mammalian Target Of Rapamycin.

Thus, the PI3K/Akt pathway is one of the most commonly deregulated signalling pathways in human cancers, contributing to tumorigenesis and metastasis. Several of the proteins involved in the PI3K-Akt-mTOR signalling pathway can function, when overexpressed, as oncoproteins, while the ones involved in quenching this pathway act as tumour suppressors.

Its deregulation is commonly associated with tumour aggressiveness and resistance to chemotherapy or radiotherapy [7]. Therefore, targeting this pathway with drug inhibitors may result in a strong and efficient anticancer effect.

Initial attempts to inhibit this signalling pathway in prostate cancer-targeted mTOR protein. All clinical trials that evaluated mTor inhibitors: Rapamycin [8], Temsirolimus [9] and Everolimus [10] were unsuccessful, with no significant antineoplastic activity.

For years, efforts have been made to develop Akt inhibitors. The research focused predominantly on the development of two separate classes: allosteric inhibitors of the Akt PH-domain and ATP-competitive inhibitors of Akt. Despite all efforts, the clinical outcomes for both classes have been disappointing. Indeed, Akt belongs to the AGC kinases family, and one of the main issues hindering drug design efforts consists of achieving selectivity over structurally similar protein kinases. The existence of three isoforms, that diverge in their affinity for ligands, function, and tissue distribution, has also hampered the development of effective Akt inhibitors [11].

Another challenge is the frequent network branching and intense cross-talk with other signalling pathways that can reverse drugs mediated inhibition effects and restore paradoxical active signalling through a high number of mechanisms.

Ultimately, the toxicity of these molecules was significant and has long worked against their development [12].

## 3. PI3K-Akt-mTor Pathway in Prostate Cancer

Increasing evidence from in vitro and animal models studies demonstrates that the PI3K-Akt-mTOR pathway plays a critical role in prostate cancer development and progression [13]. There is evidence that the deregulation of this pathway is associated with higher grade (Gleason 8–10), advanced stage (T3b-T4), and evolution to castration-resistant disease [14,15,16,17].

Several studies suggest that the Akt signalling cascade is upregulated in up to 50% of prostate cancers, through a variety of genetic alterations. Akt genetic aberrations increasing its activity have been detected in multiple malignancies but are rare events in prostate cancer (≤0.9%), whereas high-level gene amplification of Akt isoforms is more common (up to 4.5%) and positively correlates with the tumour aggressiveness [18]. In prostate cancer, the deregulation of the PI3K-Akt-mTOR pathway may be mainly driven by PTEN loss of function [19].

PTEN is a protein phosphatase that has been shown to negatively regulate this pathway by dephosphorylating PIP3 back to PIP2 (Figure 1). PTEN somatic mutations are described in many human cancers and represent the most common cause of activation of the Akt signalling pathway. Multiple mechanisms can impair PTEN activity, including homozygous deletions, somatic mutations, epigenetic or post-transcriptional modifications [20].

Its loss occurs in approximately 20% of primary prostate cancer and 50% of castration-resistant prostate tumours, depending on the study population [21].

Fluorescence in situ hybridisation (FISH) is a quantitative and highly specific method for the determination of gene copy numbers within interphase cells in tissue sections. It has long been used to detect genomic deletions of the PTEN locus. Since different mechanisms can lead to PTEN loss in prostate cancer, FISH may be underestimating the frequency of loss of PTEN [22]. Immunohistochemistry (IHC) is a less expensive and time-consuming alternative that determines the overall cellular PTEN protein expression and that could be used in routinely processed clinical pathology specimens [23]. Many protocols have been successfully validated and good concordance has been demonstrated between FISH detection of PTEN gene deletions and PTEN protein expression by IHC [23,24]. Overall, initial screening, in human prostate tissue, for PTEN loss by IHC followed by FISH analysis in ambiguous or indeterminate cases constitute the most time-effective and cost-effective protocol [25].

Pre-clinical and clinical data suggest that tumours with PTEN loss are more sensitive to AKT inhibition and support PTEN loss as a predictive factor of response to therapies targeting the Akt pathway [26]. A reciprocal relationship has been demonstrated between androgen receptor (AR) and Akt pathways in preclinical prostate cancer models with PTEN loss, such that inhibition of one leads to up-regulation of the other [21,27].

Key actors of the Akt pathway are directly involved in AR’s expression and transcriptional activity [28]. It has been demonstrated that AR phosphorylation and activation by Akt occurs predominantly at low androgen concentrations, suggesting an important role of Akt in cell growth promotion in the castrate state [29]. Indeed, the activation of the PI3K-Akt-mTOR pathway induced by PTEN loss and anti-AR treatments may promote prostate cancer cells proliferation and survival in androgen-reduced conditions. Conversely, in vitro studies have shown an AR-mediated activation of mTOR independently of PI3K-Akt stimulation in prostate cancer cells stimulated with Dihydrotestosterone (DHT) [30]. Recent discoveries indicate that the complex crosstalk between these two pathways promotes cancer progression and influences the sensitivity of prostate cancer cells to Akt inhibitors and androgen-deprivation therapies. Dual pathway inhibition may therefore result in a synergistic antitumor activity [31].

## 4. Akt Inhibitors in Prostate Cancer

Based on the elements outlined above and given the activation of the PI3K-Akt-mTOR pathway associated with a crosstalk with the AR pathway in metastatic Castration-Resistant Prostate Cancer (mCRPC), several clinical trials assessed the safety and efficacy of different Akt inhibitors combined with androgen deprivation therapies. We display herein the main outcomes of these studies.

Ipatasertib is an orally bioavailable inhibitor of all three Akt isoforms. It has been evaluated in a phase II randomised clinical trial, comparing Ipatasertib vs. placebo in Abiraterone-treated patients with mCRPC, with and without PTEN loss [32]. All patients had previously received Docetaxel and were randomised to three arms: Ipatasertib 200 mg, Ipatasertib 400 mg, or placebo, all combined with Abiraterone 1000 mg orally, once daily. Ipatasertib 400 mg showed a trend to increased radiological PFS (median 8.18 vs. 6.37 months; HR = 0.75; *p* = 0.17) and increased OS (median 18.92 months vs. 15.64 months; HR = 0.72; *p* = 0.22), compared to placebo. Patients with PTEN loss had a superior radiological PFS benefit versus those without. Adverse events (AEs) were consistent with those reported in the PI3K-Akt-mTOR pathway inhibitor class and included nausea, vomiting, diarrhoea, rash, asthenia, hyperglycemia and decreased appetite. Proportions of grade ≥3 AEs were 50.6%, 64.3%, and 35.4% in the Ipatasertib 200 mg, Ipatasertib 400 mg and placebo groups, respectively. These AEs were dose dependent and did not impact treatment dose intensity. AEs that led to discontinuation of the treatment occurred in 7 (8.0%) and 10 (11.9%) patients in the Ipatasertib 200 and 400 mg cohorts, respectively; none occurred with placebo.

Results from IPATential150, the first phase III randomised double-blind trial, involving an AKT inhibitor in prostate cancer have been recently published [33]. This study compared Ipatasertib vs. Placebo, both combined with Abiraterone in mCRPC as a first line treatment. Patients were randomised 1:1 to receive Ipatasertib 400 mg + Abiraterone 1000 mg, once daily + Prednisone 5 mg, twice daily or Placebo + Abiraterone + Prednisone. Investigator-assessed radiographic (r) PFS, according to RECIST version 1.1 or PCWG3 criteria, in patients with PTEN-loss tumours and the overall ITT population, were the coprimary endpoints. Compared to placebo, Ipatasertib showed a significantly improved rPFS and antitumor activity in patient with PTEN loss (median 18.5 vs. 16.5 months, HR = 0.77; *p* = 0.0335).

Median rPFS, in the intention to treat population, was 19.2 months in the Ipatasertib-Abiraterone group and 16.6 months in the Placebo-Abiraterone group (HR = 0.84; *p* = 0.0431, statistical significance set at α = 0.01). Secondary endpoints of confirmed objective response, PSA response, and time to PSA progression all favoured the Ipatasertib-Abiraterone arm. Secondary endpoints of confirmed objective response, PSA response, and time to PSA progression all favoured the Ipatasertib-Abiraterone arm.

In the primary analysis, tumour PTEN status was centrally assessed using a validated IHC assay. In secondary and exploratory analyses, PTEN status or PIK3CA/AKT1/PTEN alterations were detected using next-generation sequencing. rPFS was also significant in this population (median 19.1 vs. 14.2 months, HR = 0.65; *p* = 0.0206). In the ITT population the median rPFS was not statistically significant (19.2 vs. 16.6 months, HR = 0.84; *p* = 0.0431, α = 0.01). There was a 76% concordance between the two assays and, 91% of samples that had PTEN loss by next-generation sequencing were also classified as having PTEN loss by IHC.

Among patients receiving Ipatasertib, Abiraterone Acetate and Prednisone, skin rash and diarrhoea were the predominant severe toxicities. There were more grade 3–4 AEs in the Ipatasertib group compared to the placebo group. The most common grade 3–4 AEs in the Ipatasertib-Abiraterone group were rash (16%), aminotransferase increase (16%), hyperglycaemia (14%) and diarrhoea (10%). Treatment related deaths occurred in two (<1%) patients in the Placebo–Abiraterone group (lower respiratory tract infection and acute myocardial infarction) and two (<1%) patients in the Ipatasertib–Abiraterone group (chemical pneumonitis and hyperglycaemia).

Capivasertib is another highly selective pan-AKT inhibitor that has been evaluated in a phase I dose-escalation study in combination with Enzalutamide in patients with mCRPC [34]. The phase II dose identified for Capivasertib was 400 mg. The most common grade ≥3 AEs were rash (20%) and hyperglycemia (26.7%). Three patients among 12 had a composite response that was defined as PSA decline ≥50%, radiological response and/or circulating tumour cell conversion. Responses occurred in patients with PTEN loss or AKT activating mutations. ProCAID, a phase II placebo-controlled randomised trial, evaluated Capivasertib associated with Docetaxel and Prednisolone in metastatic mCRPC [35]. Patients received up to ten 21-day cycles of docetaxel (75 mg/m^2^ the first day) and prednisolone (5 mg twice daily, orally, from day 1 to day 21) and were randomly assigned to receive either Capivasertib 320 mg (orally, twice daily), or placebo, until the progression of the disease. The primary endpoint was the composite progression-free survival (cPFS) that included PSA progression events. Added to the chemotherapy, Capivasertib did not extend cPFS (median 7.03 vs. 6.70 months; HR = 0.92; *p* = 0.32) irrespective of PI3K-AKT-PTEN pathway activation status. However, there was an increased OS (secondary endpoint) with Capivasertib vs. placebo (median 31.15 vs. 20.27 months; HR = 0.54; *p* = 0.01). The most common adverse events of any grade related to Capivasertib were nausea, diarrhoea, rash, and fatigue (Table 1).

## 5. Ongoing Trials

Several ongoing trials assess several Akt inhibitors in prostate cancer with different combinations, at different stages. These trials are summarised in Table 2 [36].

Phase I and II studies are evaluating Ipatasertib safety, tolerability, pharmacokinetics and efficacy in combination with Hydroxychloroquine, PARP inhibitors or immunotherapies in patients with metastatic castration-resistant prostate cancer.

Trials are also ongoing at earlier stages of the disease. A phase III double-blind randomised placebo-controlled study now compares Capivasertib vs. placebo in patients with de novo metastatic hormone-sensitive prostate cancer characterised by PTEN deficiency, treated with abiraterone plus prednisone.

## 6. Conclusions

PI3K-AKT-mTOR is an important signalling pathway of cellular metabolism. Its deregulation has been shown to play a critical role in many cancers, particularly through PTEN alterations. In prostate cancer, a crosstalk with the AR pathway has been demonstrated, which may participate in resistance to castration and escape from hormone therapy. Preliminary clinical studies investigated the combined blockade of these two pathways and have shown encouraging results in terms of radiologic PFS in mCRPC, more markedly in patients with PTEN loss, with a tolerance that appeared to be acceptable. Several trials based on these initial findings are ongoing. Future challenges will be to identify markers that will allow a better selection of patients who can benefit the most from Akt inhibitors, possibly at earlier stages of the disease.

## Figures and Tables

**Table 1:** IPATASERTIB (IPATential150) versus CAPIVASERTIB (ProCAID) in prostate cancer.

	IPATASERTIB	CAPIVASERTIB
Clinical trial	IPATential	ProCAID
Phase	III	II
Number of patients enrolled	1101	150
Primary endpoint	rPFS	cPFS
Stage of the disease	mCRPC	mCRPC
Association to AKTi	Abiraterone + Prednisolone	Docetaxel + Prednisolone
Control	Placebo	Placebo
Primary outcome’s HR	0.77 (95% CI, 0.61–0.98); *p* = 0.034	0.92 (80% CI, 0.73–1.16); *p* = 0.32
Grade ≥ 3 adverse events	70%	62%
AEs leading to treatment discontinuation	21%	23%

rPFS: radiological progression-free survival; cPFS: composite radiological progression-free survival; AKTi: AKT inhibitor; mCRPC: metastatic castration resistant prostate cancer, HR: Hazard Ratio; AEs: adverse events.

**Table 2:** Ongoing trials in Prostate Cancer using Akt inhibitors.

Drug	Phase	NCT Number	Conditions	Investigator	Regimen	Status	Title of the Study	Primary Outcome
Capivasertib	III	NCT04493853	De novo metastatic hormone-sensitive prostate cancer with PTEN deficiency	AstraZeneca	Capivasertib + Abiraterone	Recruiting	A double-blind, randomised, placebo-controlled study assessing the efficacy and safety of Capivasertib + Abiraterone versus placebo+abiraterone as a treatment for patients with denovo metastatic hormone-sensitive prostate cancer characterised by PTEN deficiency.	Radiographic progression-free survival (rPFS).
Capivasertib (AZD5363)	I	NCT04087174	Metastatic castration-resistant prostate cancer	AstraZeneca Parexel	Cabivasertibe + Enzalutamide or Abiraterone	Completed	Open-label, multi-centre study to assess the safety, tolerability, and pharmacokinetics of Capivasertib (AZD5363) in combination with novel agents in patients with metastatic castration resistant prostate cancer.	Number of patients with dose-limiting toxicity and number of patients with adverse events.
MK2206	I	NCT01480154	Solid neoplasm, melanoma, prostate and kidney cancers	Jyoti Malhotra (Rutgers Cancer Institute of New Jersey)	Akt inhibitor MK2206 + Hydroxychloroquine	Active, not recruiting	Akt inhibitor MK2206 and hydroxychloroquine in treating patients with Advanced solid tumours, melanoma, prostate or kidney cancer.	To define the maximum tolerated dose of MK-2206 and hydroxychloroquine when used in combination.
Ipatasertib (GDC-0068)	Ib/II	NCT01485861	Castration-resistant prostate cancer previously treated with Docetaxel	Genentech, Inc.	Ipatasertibe or Apitolisilib + Abiraterone	Active, not recruiting	Ipatasertib (GDC-0068) or Apitolisib (GDC-0980) with Abiraterone Acetate versus Abiraterone Acetate in patients with castration-resistant prostate cancer previously treated with Docetaxel-based chemotherapy.	Recommended phase II dose of Ipatasertib, percentage of radiographic progression and progression free survival with or without PTEN loss.
Ipatasertib	Ib	NCT04404140	Metastatic castration-resistant prostate cancer	Hoffmann-La Roche	Ipatasertib + Atezolizumab + Docetaxel	Recruiting	A multicentre study evaluating the safety, efficacy and pharmacokinetics of Ipatasertib In combination with Atezolizumab and Docetaxel in metastatic castration-resistant prostate cancer.	Percentage of patients with adverse events, confirmed PSA response, overall response rate.
Ipatasertib	I	NCT04737109	Breast, ovarian and prostate cancers	Hoffmann-La Roche	Ipatasertib + Rucaparib	Active, not recruiting	A multicentre study evaluating the safety and efficacy of Ipatasertib in combination with Rucaparib in patients with advanced breast, ovarian, or prostate cancer.	Percentage of patients with adverse events, maximum-dose tolerated of the Ipatersertib and Rucaparib combination, percentage of patients with PSA response
Ipatasertib	I	NCT03673787	Solid tumour, glioblastoma, metastatic prostate cancer	Juanita Lopez (National Health Service, UK)	Ipatasertib + Atezolizumab	Recruiting	Ipatasertib in combination with Atezolizumab in patients with advanced solid tumours with PI3K pathway hyperactivation.	To determine the maximum tolerated dose in Phase I. Number and type of treatment-related adverse events of the two drugs combination.
Ipatasertib	I/II	NCT04737109	Localised high-risk prostate cancer	David VanderWeele (Northwestern University)	Ipatasertib + Darolutamide	Recruiting	Neoadjuvant androgen deprivation, Darolutamide, and Ipatasertib in men with localised, high-risk prostate cancer.	Pathological Complete Response Rate

## Data Availability

Not applicable.
